# A Convenient Laboratory‐Scale Synthesis Route Toward the PPARα Agonist Pemafibrate

**DOI:** 10.1002/open.202600009

**Published:** 2026-03-25

**Authors:** Felien Morlion, Dorien Clarisse, Karolien De Bosscher, Matthias D’hooghe

**Affiliations:** ^1^ SynBioC Research Group Department of Green Chemistry and Technology Ghent University Ghent Belgium; ^2^ Translational Nuclear Receptor Research VIB‐UGent Center for Medical Biotechnology Ghent Belgium; ^3^ Department of Biomolecular Medicine Ghent University Ghent Belgium; ^4^ Cancer Research Institute Ghent (CRIG) Ghent Belgium

**Keywords:** cost‐efficient, optimization, pemafibrate, synthetic methods, total synthesis

## Abstract

Pemafibrate is a potent and selective agonist of peroxisome proliferator‐activated receptor alpha (PPARα). It was designed to improve lipid metabolism with enhanced safety and efficacy compared to traditional fibrates and is clinically approved in Japan to treat hyperlipidemia. This strong PPARα agonist is also widely used in academic research to study various metabolic and cardiovascular disorders. However, the very high commercial cost can limit academic studies when gram‐scale quantities are required or when structural modifications are being explored *en route* to novel applications. In this work, convenient and high‐yielding laboratory‐scale procedures are presented for the synthesis of pemafibrate. With a primary focus on practicality and efficiency, literature and patent protocols were adapted, optimized, and combined to establish a new, straightforward, and efficient six‐step synthetic route that starts from easily accessible and cheap substrates toward enantiopure pemafibrate at the multigram scale.

## Graphical abstract

Pemafibrate is a potent peroxisome proliferator‐activated receptor alpha (PPARα) agonist that is widely used in metabolic disease research. An optimized, practical, and high‐yielding synthetic route toward pemafibrate in excellent optical purity is reported. This multigram‐scale approach overcomes limitations associated with previously reported methods and considerably lowers costs for academic studies.

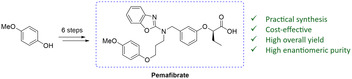

## 1 Introduction

Peroxisome proliferator‐activated receptors (PPARs) are transcription factors belonging to the nuclear receptor superfamily and play a crucial role in regulating lipid and glucose metabolism, energy homeostasis, and inflammation. PPARα, one of the three PPAR isoforms, not only regulates fatty acid uptake and oxidation but also modulates inflammatory pathways and vascular function. This makes PPARα a relevant therapeutic target for the treatment of metabolic disorders such as metabolic dysfunction‐associated steatotic liver disease, type 2 diabetes, and atherosclerosis [1, 2, 3, 4].

Traditional fibrates such as fenofibrate, bezafibrate, and gemfibrozil are PPARα agonists that have been used for over 50 years to lower blood triglyceride levels and improve cardiovascular health. However, the potency and isoform selectivity of first‐generation fibrates is low, resulting in dose‐dependent adverse effects such as myopathy, liver dysfunction, and rhabdomyolysis [4, 5]. To overcome the limitations associated with traditional fibrates, selective PPARα modulators (SPPARMα) have been developed to enhance lipid‐lowering efficacy while reducing adverse effects. Pemafibrate (Parmodia, K‐877, (*R*)‐K‐13675) is a highly potent SPPARMα, showing >2500‐fold stronger PPARα activation compared to fenofibric acid, and has >1000 times higher selectivity for PPARα over PPARγ and PPARβ/δ. Pemafibrate was approved in Japan in 2017 for the treatment of hyperlipidemia and continues to be the topic of advanced research related to other metabolic, inflammatory, and cardiovascular disorders [6, 7, 8, 9].

However, the very high commercial cost of pemafibrate (approximately €1000 per gram in 2025) can be a limiting factor to academic research when multigram quantities are required for in vivo studies or elaborate chemical modifications *en route* to new applications. Although the synthesis of pemafibrate has been described in a few journal articles [9, 10, 11, 12] and patents [13, 14, 15, 16, 17, 18, 19, 20, 21], our attempts to apply these protocols revealed many hurdles in terms of practicality and efficiency. In addition, pemafibrate‐related patents are not all available in English, contain extensive side information, and are difficult to compare, while the pemafibrate‐related journal articles originate from early development stages and describe various approaches from small‐scale synthesis to large‐scale production. During our investigations, it became clear that these literature protocols contain potentially useful but also inefficient steps, rendering the overall applicability of each procedure to be rather low. Therefore, a thorough and detailed analysis of all reported reaction steps was performed experimentally, and the most efficient approaches were further optimized and combined in view of laboratory‐scale implementation to present a new overall strategy for pemafibrate synthesis. The enantiomeric purity of pemafibrate is typically assessed using chiral liquid chromatography‐mass spectrometry (LC‐MS) methods in the literature [11], but such analyses are often challenging to reproduce without identical chromatographic columns. Therefore, an alternative and reliable nuclear magnetic resonance (NMR)‐based analysis method was developed to evaluate the chiral purity by reacting pemafibrate with a chiral alcohol.

## 2 Results and Discussion

**SCHEME 1:**
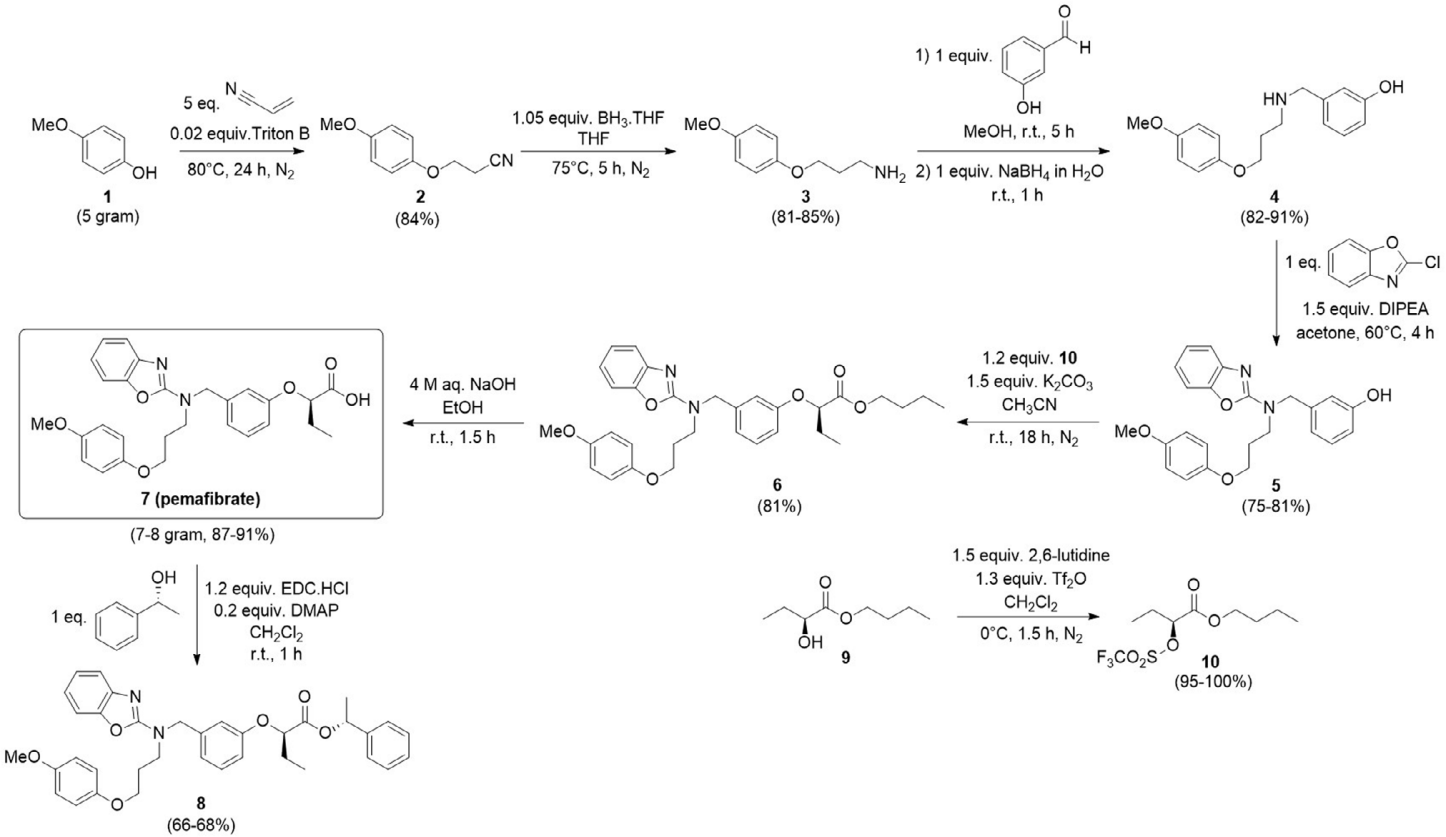
Synthesis route toward pemafibrate **7** and the corresponding (*R*)‐1‐phenylethyl ester **8** starting from 4‐methoxyphenol **1**.

The presented synthetic route toward pemafibrate **7** (Scheme 1) is mainly based on different procedures published by the Kowa Company, the inventor and manufacturer of pemafibrate [11, 12, 15]. Other methods developed by the same company were also considered but not adopted, as they were either unoptimized, lacked sufficient detail, or produced pemafibrate as a racemic mixture [9, 10, 13, 14, 16, 17]. The relevant procedures from this search were compared with other available patent literature data to identify opportunities for further optimization [18, 19, 20, 21].

Our optimized six‐step synthesis of pemafibrate **7** starts with a Michael addition of 4‐methoxyphenol **1** across acrylonitrile, affording propionitrile **2**. Literature procedures [12, 15] employed two equivalents of acrylonitrile applying various reaction times. However, under these conditions, only ~50% conversion was achieved after 24 h in our hands. To enhance conversion and yield, the amount of acrylonitrile was increased to five equivalents, resulting in 90% conversion within 24 h. Prolonged reaction times did not further improve the conversion. After extraction and recrystallization, 3‐(4‐methoxyphenoxy)propanenitrile **2** was isolated in 84% yield, slightly exceeding the previously reported yields of 76%–77% [12, 15]. Subsequent reduction of nitrile **2** to the corresponding primary amine was performed using 1.05 equivalents of BH_3_·THF. A reaction temperature of 75°C was selected to ensure full conversion. The reaction mixture was quenched with 4 M NaOH and stirred overnight at 65°C. Extraction with toluene then afforded 3‐(4‐methoxyphenoxy)propan‐1‐amine **3** in 81–85% yield, within the range of reported values of 79%–90% [12, 15].

The third reaction step involves a well‐established reductive amination of primary amine **3** with 3‐hydroxybenzaldehyde and NaBH_4_ to afford secondary amine **4**. This protocol was adopted from the literature due to its simplicity and high efficiency [12]. The latter amine **4** was then deployed to perform a nucleophilic aromatic substitution onto 2‐chlorobenzoxazole. While Kowa procedures [12, 15] employ DMF and Et_3_N to that end, an alternative patent method [18] using acetone and *N*,*N*‐diisopropylethylamine (DIPEA) was selected here, which facilitated solvent evaporation during work‐up. LC‐MS monitoring and NMR analysis confirmed comparable outcomes for both conditions. Precipitation in tetrahydrofuran (THF)/heptane (1/10), as described in the literature [18], gave tertiary amine **5** as a beige solid in 75%–81% yield, while we reached a yield of 54%–69% via a previously reported column chromatography purification method [12]. Another patent suggested to switch the reaction step order (reductive amination vs. nucleophilic aromatic substitution) to minimize side product formation. These modifications were not implemented, as they prolonged reaction times to 30 h for both steps, and the observed side products did not compromise the purity of our final pemafibrate **7** [21].

For further functionalization of phenol compound **5**, the triflate of (*S*)‐butyl 2‐hydroxybutanoate **10** had to be prepared first. Triflate formation was optimized by conducting the reaction at 0°C for 1.5 h [22] instead of at −20°C for 5 h [11], thereby simplifying temperature control. Triflate **10** was used without further purification after extraction and was obtained in a high yield of 95%–100%. Nucleophilic attack of the phenolate anion derived from compound **5** onto triflate **10** afforded pure butyl ester **6** after column chromatography in a yield of 81%. Literature procedures typically use 1.2–1.5 equivalents of triflate **10** [11, 15]. The minimal reported amount was selected to reduce reagent costs and was sufficient to reach full conversion after 18 h.

Alternative routes for the transformation of compound **3** to **6** have been described, but these procedures were not implemented because of the following limitations. One patent suggested to couple triflate **10** to 3‐hydroxybenzaldehyde, followed by reductive amination with propanamine **3** and nucleophilic aromatic substitution with 2‐chlorobenzoxazole to yield compound **6** [14]. This route was not preferred because we chose to introduce the chiral center as late as possible to minimize cost and reduce racemization risk. An alternative for compound **9** has been reported as well, but this method involved more reaction steps and lacked data on chiral purity of the produced pemafibrate **7** [20].

Finally, hydrolysis of butyl ester **6** by means of NaOH in EtOH resulted in pure pemafibrate **7** as an amorphous solid after extraction in a yield of 87%–91%. The MTBE washing step described in the literature [11] was omitted, as this complicated the extraction by forming inseparable layers. The obtained amorphous solid can be crystallized into one of the pemafibrate polymorphs to enhance its solubility and bioavailability, following the procedures described by Li et al. [23].

**FIGURE 1:**
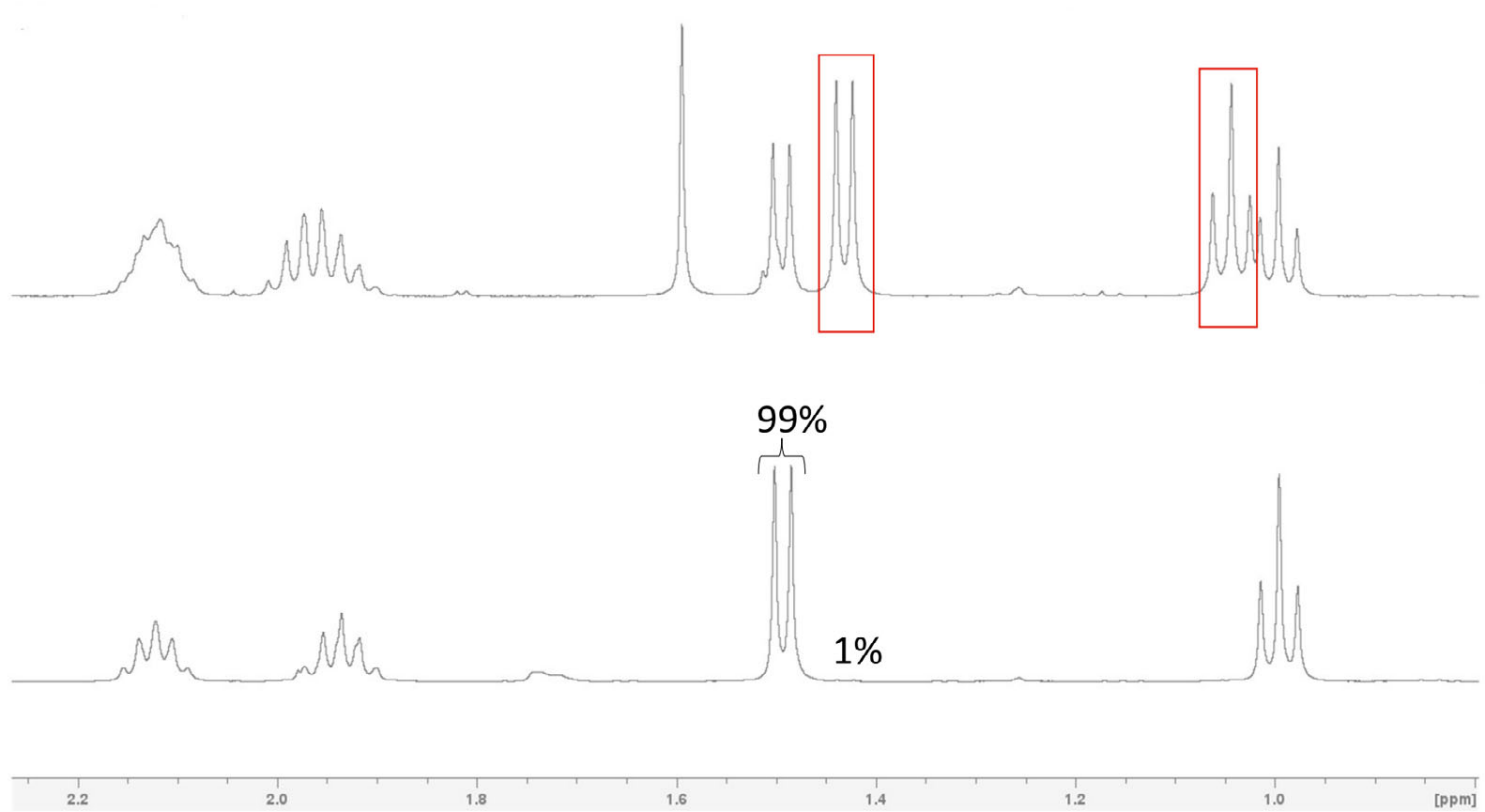
^1^H NMR spectrum of diastereomers **13** (up) and ^1^H NMR spectrum of optically pure (ee 98%) compound **8** (down). A shift of the doublet at 1.5 ppm and the triplet at 1.0 ppm pertaining to the (*R*,*R*)‐isomer is observed in the (*R*,*S*)‐isomer (red rectangles) of product **13** and is minimally present (1%, based on integration) in product **8**.

**SCHEME 2:**
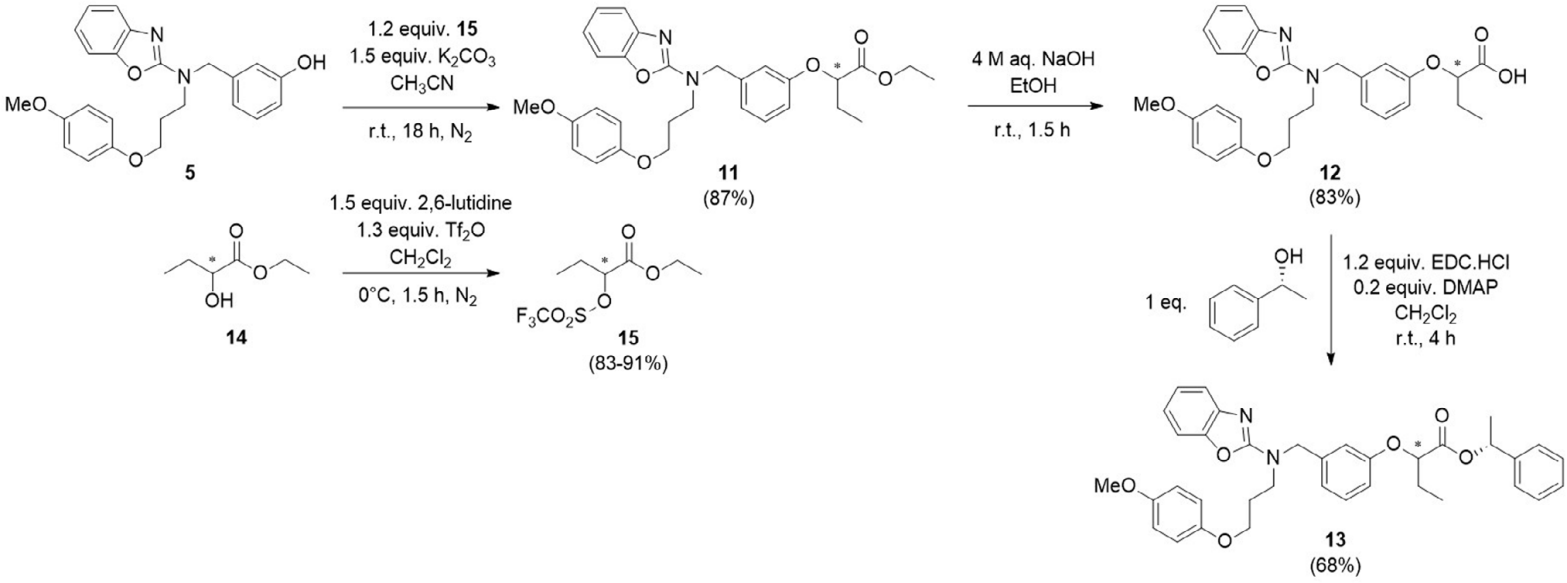
Synthesis route toward racemic pemafibrate **12** and the diastereomeric mixture of the corresponding (*R*)‐1‐phenylethyl ester **13**.

The enantiomeric purity of pemafibrate **7** was assessed by derivatization to the corresponding (*R*)‐1‐phenylethyl ester **8**, allowing detection of potential diastereomeric signals by means of ^1^H NMR analysis. Although this method is less sensitive than chiral LC‐MS, it is more reproducible without specialized chromatographic columns and provides sufficient accuracy for research purposes. For comparison and unambiguous assessment, racemic pemafibrate **12** was prepared as well through reaction of tertiary amine **5** with the triflate of racemic ethyl 2‐hydroxybutanoate **15**, followed by hydrolysis (Scheme 2). The obtained racemic pemafibrate **12** was also esterified using (*R*)‐1‐phenylethanol to the (*R*,*S*)‐ and (*R*,*R*)‐diastereomeric mixture **13** to enable comparison with the ^1^H NMR spectra of compound **8**. Integration confirmed that pemafibrate derivative **8** contained max. 1% of the (*R*,*S*)‐isomer and min. 99% of the (*R*,*R*)‐diastereomer, corresponding to an enantiomeric excess of at least 98% of the *R*‐enantiomer in the produced pemafibrate **7** (Figure 1). In this way, the enantiomeric purity of pemafibrate **7**, prepared by means of our optimized protocol, was unequivocally established.

## 3 Conclusion

Different methods for the synthesis of the potent and selective PPARα agonist pemafibrate were assessed, adapted, optimized, and combined into a new strategy to develop a convenient and effective laboratory approach toward pemafibrate. This convenient and cost‐effective new route consists of six consecutive steps and delivers the desired pemafibrate at the multigram scale in a high overall yield of 35% and in excellent enantiomeric purity.

## 4 Experimental Section

### 4.1 General

All commercially available reagents and solvents were used without prior purification. NMR spectra (^1^H NMR, ^19^F NMR, ^13^C{^1^H} NMR) were recorded at 400, 376.5 and 100.6 MHz, respectively, on a Bruker Avance III HD spectrometer equipped with a 1H/BB z‐gradient probe (BBO, 5 mm) and using CDCl_3_ or alternative deuterated solvents and trimethyl silane as an internal standard. Spectra were viewed and processed with TOPSPIN 4.3.0 software. HPLC‐MS analysis was conducted on an Agilent 1200 Series HPLC system, equipped with a Supelco Ascentic Express C18 column (3 cm × 4.6 mm, 2.7 µm fused‐core particles, 90 Å), coupled to an Agilent 1100 Series MS with electrospray ionization (70 eV) with a single quadrupole detector. Automated chromatography was performed using Büchi Pure C‐815 Flash chromatography system with UV (200–400 nm) detection and an evaporative light scattering detector. FTIR spectra were acquired using a Shimadzu IRAffinity‐1S with ATR crystal and analyzed via LabSolutions IR software. TLC analysis was conducted with Merck Silica Gel 60 F_254_ plates. Anhydrous solvents were supplied by a MBraun SPS‐800 solvent purification system.

### 4.2 Synthetic Procedures

#### 4.2.1 3‐(4‐Methoxyphenoxy)propanenitrile (2)

4‐Methoxyphenol **1** (5.000 g, 40.28 mmol, 1 equiv.) was dissolved in acrylonitrile (10.686 g, 201.39 mmol, 5 equiv.) under a nitrogen atmosphere, and 0.35 mL of Triton B (40% in MeOH, w/w) (0.77 mmol, 0.02 equiv.) was added at room temperature. The mixture was stirred at 80°C for 24 h. The resulting reaction mixture was diluted with EtOAc (20 mL), and the organic layer was washed sequentially with 20 mL of 5% aq. NaOH, H_2_O, 1 M HCl, H_2_O, sat. NaHCO_3_, and brine. The organic layer was dried with MgSO_4_ and concentrated *in vacuo*. The residue was dissolved in EtOAc (5 mL), hexane (25 mL) was added, and the flask was shaken. The resulting precipitation was filtered off and to give 3‐(4‐methoxyphenoxy)propanenitrile **2** (5.988 g, 33.79 mmol) as beige‐gray crystals in a yield of 84%. Procedure adapted from the literature [12]; spectral data corresponded to those found in the literature [12].

#### 4.2.2 3‐(4‐Methoxyphenoxy)propylamine (3)

3‐(4‐Methoxyphenoxy)propanenitrile **2** (5.988 g, 33.79 mmol, 1 equiv.) was dissolved in anhydrous THF (25 mL) under a nitrogen atmosphere, and the mixture was heated to 65°C. After dropwise addition of BH_3_·THF (1.0 mol/L in THF, 35.48 mL, 35.48 mmol, 1.05 equiv.), the mixture was stirred at 75°C for 5 h. The solution was cooled to 0°C and 4 M NaOH (40 mL) was added slowly. The resulting mixture was stirred at 65°C for 16 h and cooled to room temperature, after which toluene (120 mL) was added. The organic layer was washed with H_2_O (3 × 75 mL) and brine (75 mL), dried with MgSO_4_, and concentrated to give 3‐(4‐methoxyphenoxy)propylamine **3** (5.218 g, 28.79 mmol, 85%) as an off‐white solid. Procedure adapted from the literature [12]; spectral data corresponded to those found in the literature [12].

#### 4.2.3 3‐{[(3‐(4‐Methoxyphenoxy)propyl)amino]methyl}phenol (4)

3‐Hydroxybenzaldehyde (3.516 g, 28.79 mmol, 1 equiv.) was added to a solution of 3‐(4‐methoxyphenoxy)propylamine **3** (5.218 g, 28.79 mmol, 1 equiv.) in methanol (25 mL) in a 250 mL flask at room temperature, and the mixture was stirred for 5 h. A solution of NaBH_4_ (1.089 g, 28.79 mmol, 1 equiv.) in H_2_O (50 mL) was added slowly at 0°C, and the mixture was stirred at room temperature for 1 h when gas evolution had stopped. The obtained white solids were filtered, washed with H_2_O (30 mL), and dried under reduced pressure at 50°C to result in 7.561 g of 3‐{[(3‐(4‐methoxyphenoxy)propyl)amino]methyl}phenol **4** (26.31 mmol, 91%). Procedure adapted from the literature [12]; spectral data corresponded to those found in the literature [12].

#### 4.2.4 3‐{[Benzo[*d*]oxazol‐2‐yl(3‐(4‐methoxyphenoxy)propyl)amino]methyl}phenol (5)

Secondary amine **4** (7.561 g, 26.31 mmol, 1 equiv.) was dissolved in acetone (75 mL) and DIPEA (5.101 g, 39.47 mmol, 1.5 equiv.) was added. 2‐Chlorobenzoxazole (4.040 g, 26.31 mmol, 1 equiv.) was added dropwise, and the mixture was stirred for 15 min at room temperature. Then, the temperature was raised to 60°C, and the reaction mixture was stirred for 4 h, after which the solvent was removed *in vacuo*. EtOAc (75 mL) was added to the residue, and the solution was washed with 1N HCl (2 x 50 mL), dried with MgSO_4_ and concentrated under vacuum. The resulting yellow‐brown oil was dissolved in 7.5 mL of THF, and 75 mL of heptane was slowly added. After 15 h of stirring, the resulting beige solid was filtered off and dried under vacuum to yield tertiary amine **5** (8.596 g, 21.25 mmol, 81%). Procedure adapted from the literature [18]; spectral data corresponded to those found in the literature [12].

#### 4.2.5 Butyl (*S*)‐2‐{[(trifluoromethyl)sulfonyl]oxy}butanoate (10)

A solution of (*S*)‐butyl 2‐hydroxybutanoate **9** (5 g, 31.21 mmol, 1 equiv.) and 2,6‐lutidine (5.016 g, 46.81 mmol, 1.5 equiv.) in dry CH_2_Cl_2_ (120 mL) was cooled to 0°C under a nitrogen atmosphere. Trifluoromethanesulfonic anhydride (11.447 g, 40.57 mmol, 1.3 equiv.) was added dropwise over 20 min. The resulting mixture was stirred at 0°C for 1.5 h and was then washed with 3/1 mixtures of brine and 1N HCl (3 × 250 mL), dried with MgSO_4_, and concentrated under reduced pressure to afford triflate **10** (8.625 g, 29.51 mmol, 95%) as a light‐yellow oil. Procedure adapted from the literature [22]; spectral data corresponded to those found in the literature [22].

#### 4.2.6 Butyl (*R*)‐2‐{3‐[(benzo[*d*]oxazol‐2‐yl(3‐(4‐methoxyphenoxy)propyl)amino)methyl]phenoxy}butanoate (6)

A solution of triflate **10** (7.454 g, 25.50 mmol, 1.2 equiv.) in CH_3_CN (16 mL) was added over 5 min to a mixture of product **5** (8.596 g, 21.25 mmol, 1 equiv.) and K_2_CO_3_ (4.406 g, 31.88 mmol, 1.5 equiv.) in CH_3_CN (160 mL) under a nitrogen atmosphere. The resulting mixture was stirred at room temperature for 18 h. The solids were filtered off and the filtrate was evaporated under reduced pressure. The residue was redissolved in EtOAc (80 mL), washed with H_2_O (80 mL) and brine (80 mL), dried over MgSO_4_, concentrated *in vacuo*, and purified by normal phase flash chromatography (EtOAc/hexane, 0/100 → 30/70) to yield butyl ester **6** as a yellow oil (9.451 g, 17.29 mmol, 81%). Procedure adapted from the literature [11]; spectral data corresponded to those found in the literature [11].

#### 4.2.7 Pemafibrate (7)

Butyl ester **6** (9.451 g, 17.29 mmol, 1 equiv.) was dissolved in EtOH (40 mL), and 4 M NaOH (7 mL) was added at 0°C. The reaction mixture was stirred at room temperature for 1.5 h, and the solvent was evaporated. The residue was redissolved in water (50 mL), acidified with 37% HCl until pH 1 at 0°C, and extracted with EtOAc (50 mL). The organic layer was washed with brine (50 mL), dried with MgSO_4_ and concentrated under vacuum to yield pemafibrate **7** (7.691 g, 15.68 mmol, 91%) as a clear colorless solid. Procedure adapted from the literature [11]; spectral data corresponded to those found in the literature [11].

#### 4.2.8 (*R*)‐1‐Phenylethyl (*R*)‐2‐{3‐[(benzo[*d*]oxazol‐2‐yl(3‐(4‐methoxyphenoxy)propyl)amino)methyl]phenoxy}butanoate (8)

Pemafibrate **7** (60 mg, 0.122 mmol, 1 equiv.) and (*R*)‐(+)‐1‐phenylethanol (14.9 mg, 0.122 mmol, 1 equiv., ee > 97%), were dissolved in dry CH_2_Cl_2_ (1 mL) under a nitrogen atmosphere at 0°C. EDC·HCl (28.1 mg, 0.147 mmol, 1.2 equiv.) and DMAP (3.0 mg, 0.024 mmol, 0.02 equiv.) were added, and the mixture was stirred for 1.5 h until conversion was complete. The reaction mixture was diluted with CH_2_Cl_2_ (15 mL), and the organic phase was washed with H_2_O (3 × 10 mL), dried over MgSO_4_, and evaporated. The colorless oil was purified with normal phase column chromatography (EtOAc/hexane, 0/100 → 40/60) to yield compound **8** as a clear colorless oil (49.6 mg, 0.083 mmol, 68%).

R_f_ = 0.74 (EtOAc/hexane 7/3). 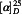 = 63.6° (*c* = 0.12, CHCl_3_). ^**1**^ **H NMR** (400 MHz, CDCl_3_): *δ* 1.00 (3H, t, *J* = 7.4 Hz); 1.49 (3H, d, *J* = 6.6 Hz); 1.90–1.98 (2H, m); 2.12 (2H, quint, *J* = 6.5 Hz); 3.65 (2H, t, *J* = 7.0 Hz); 3.75 (3H, s); 3.94 (2H, t, *J* = 5.9 Hz); 4.55 (1H, t, *J* = 6.1 Hz); 4.69 (1H, d, *J* = 15.8 Hz); 4.74 (1H, d, *J* = 15.8 Hz); 5.89 (1H, q, *J* = 6.6 Hz); 6.75 (1H, d x d, *J* = 8.5, 2.3 Hz); 6.79–6.83 (5H, m); 6.89 (1H, br. d, *J* = 7.5 Hz); 7.00 (1H, t x d, *J* = 7.7, 0.9 Hz); 7.13–7.25 (8H, m); 7.37 (1H, d, *J* = 7.7 Hz). ^**13**^ **C NMR** (100.6 MHz, CDCl_3_): *δ* 9.6; 22.0; 26.1; 27.5; 45.1; 52.1; 55.7; 65.6; 73.2; 77.5; 108.8; 114.0; 114.6; 114.7 (2 x C); 115.5 (2 x C); 116.2; 120.4; 120.7; 124.0; 126.0 (2 x C); 127.9; 128.4 (2 x C); 129.8; 138.7; 140.9; 143.5; 148.9; 152.9; 153.9; 158.3; 162.7; 170.8. **IR** (cm^−1^): ν_max_ = 2318, 1746, 1639, 1578, 1508, 1460, 1231. **MS** (70 eV): m/z (%) 595 (M^+^ + 1, 100).

#### 4.2.9 Ethyl 2‐{[(trifluoromethyl)sulfonyl]oxy}butanoate (15)

A solution of racemic ethyl‐2‐hydroxybutanoate **14** (211.0 mg, 1.597 mmol, 1 equiv.) and 2,6‐lutidine (256.6 mg, 2.395 mmol, 1.5 equiv.) in dry CH_2_Cl_2_ (5 mL) was cooled to 0°C under a nitrogen atmosphere. Trifluoromethanesulfonic anhydride (585.6 mg, 2.076 mmol, 1.3 equiv.) was added dropwise over 20 min. The resulting mixture was stirred at 0°C for 1.5 h and was then diluted with CH_2_Cl_2_ (10 mL), washed with 3/1 mixtures of brine and 1N HCl (3 × 15 mL), dried with MgSO_4_, and concentrated under reduced pressure to afford triflate **15** (385.2 mg, 1.458 mmol, 91%) as a light‐orange oil.

R_f_ = 0.41 (EtOAc/hexane 3/7). ^**1**^ **H NMR** (400 MHz, CDCl_3_): *δ* 1.06 (3H, t, *J* = 7.4 Hz); 1.32 (3H, t, *J* = 7.2 Hz); 1.97–2.14 (2H, m); 4.24–4.36 (2H, m); 5.07 (1H, d x d, *J* = 7.3, 4.6 Hz). ^**19**^ **F NMR** (376.5 MHz, CDCl_3_): *δ* −75.0 (3 x F, s). ^**13**^ **C NMR** (100.6 MHz, CDCl_3_): *δ* 8.9; 14.0; 25.5; 62.6; 84.8; 118.5 (q, ^1^ *J*_*C*‐F_ = 319.4 Hz); 167.0. **IR** (cm^−1^): ν_max_ = 1759, 1416, 1198, 1144, 962, 945, 611.

#### 4.2.10 Ethyl 2‐{3‐[(benzo[*d*]oxazol‐2‐yl(3‐(4‐methoxyphenoxy)propyl)amino)methyl]phenoxy}butanoate (11)

A solution of triflate **15** (372.4 mg, 1.409 mmol, 1.2 equiv.) in CH_3_CN (1 mL) was added over 5 min to a mixture of product **5** (475.0 mg, 1.174 mmol, 1 equiv.) and K_2_CO_3_ (243.5 mg, 1.762 mmol, 1.5 equiv.) in CH_3_CN (10 mL) under a nitrogen atmosphere. The resulting mixture was stirred at room temperature for 18 h. The solids were filtered off, and the filtrate was evaporated under reduced pressure. The residue was redissolved in EtOAc (20 mL), washed with H_2_O (20 mL) and brine (20 mL), dried over MgSO_4_, concentrated *in vacuo*, and purified by normal phase flash chromatography (EtOAc/hexane, 0/100 → 40/60) to yield ethyl ester **11** as a light‐yellow oil (532.3 mg, 1.026 mmol, 87%).

R_f_ = 0.69 (EtOAc/hexane 7/3). ^**1**^ **H NMR** (400 MHz, CDCl_3_): *δ* 1.05 (3H, t, *J* = 7.4 Hz); 1.17 (3H, t, *J* = 7.1 Hz); 1.96 (2H, quint, *J* = 7.1 Hz); 2.14 (2H, quint, *J* = 6.5 Hz); 3.70 (2H, t, *J* = 7.1 Hz); 3.76 (3H, s); 3.96 (2H, t, *J* = 6.0 Hz); 4.07–4.20 (2H, m); 4.51 (1H, t, *J* = 6.2 Hz); 4.72 (1H, d, *J* = 15.8 Hz); 4.77 (1H, d, *J* = 15.8 Hz); 6.77 (1H, d x d, *J* = 8.0, 2.4 Hz); 6.81 (4H, br. s); 6.85–6.86 (1H, m); 6.90 (1H, d, *J* = 7.6 Hz); 7.01 (1H, t x d, *J* = 7.6, 1.0 Hz); 7.16 (1H, t x d, *J* = 7.7, 1.1 Hz); 7.19–7.23 (2H, m); 7.37 (1H, d x d, *J* = 7.8, 0.5 Hz). ^**13**^ **C NMR** (100.6 MHz, CDCl_3_): *δ* 9.6; 14.1; 26.1; 27.6; 45.2; 52.1; 55.7; 61.1; 65.6; 77.7; 108.7; 113.9; 114.7 (3 x C); 115.5 (2 x C); 116.2; 120.4; 120.7; 123.9; 129.8; 138.7; 143.5; 148.9; 152.8; 153.9; 158.4; 162.7; 171.5. **IR** (cm^−1^): ν_max_ = 2318, 1749, 1638, 1578, 1508, 1458, 1231. **MS** (70 eV): m/z (%) 519 (M^+^ + 1, 100).

#### 4.2.11 2‐{3‐[(Benzo[*d*]oxazol‐2‐yl(3‐(4‐methoxyphenoxy)propyl)amino)methyl]phenoxy}butanoic acid (racemic pemafibrate (12)

Ethyl ester **11** (89.2 mg, 0.153 mmol, 1 equiv.) was dissolved in EtOH (0.5 mL), and 4 M NaOH (0.07 mL) was added at 0°C. The reaction mixture was stirred at room temperature for 1.5 h, and the solvent was evaporated. The residue was redissolved in water (15 mL), acidified with 37% HCl until pH 1 at 0°C, and extracted with EtOAc (15 mL). The organic layer was washed with brine (15 mL), dried with MgSO_4_ and concentrated under vacuum to yield racemic pemafibrate **12** (62.8 mg, 0.128 mmol, 83%) as a clear colorless solid. Spectral data corresponded to those found in the literature [11].

#### 4.2.12 (*R*)‐1‐Phenylethyl 2‐{3‐[(benzo[*d*]oxazol‐2‐yl(3‐(4‐methoxyphenoxy)propyl)amino)methyl]phenoxy}butanoate (13)

Racemic pemafibrate **12** (43.0 mg, 0.088 mmol, 1 equiv.) and (*R*)‐(+)‐1‐phenylethanol (10.7 mg, 0.088 mmol, 1 equiv., ee > 97%), were dissolved in dry CH_2_Cl_2_ (1 mL) under a nitrogen atmosphere at 0°C. EDC·HCl (20.2 mg, 0.105 mmol, 1.2 equiv.) and DMAP (2.1 mg, 0.018 mmol, 0.2 equiv.) were added and the mixture was stirred for 4 h, until conversion was complete. The reaction mixture was diluted with CH_2_Cl_2_ (15 mL), and the organic phase was washed with H_2_O (3 × 10 mL), dried over MgSO_4_ and evaporated. The colorless oil was purified with normal phase column chromatography (EtOAc/hexane, 0/100 → 40/60) to yield diastereomer **13** as a clear colorless oil (35.5 mg, 0.060 mmol, 68%).

Spectral data derived from a mixture of two diastereomers (*dr* = 42/58, (*R*,*R*)/(*R*,*S*)).

R_f_ = 0.71 (EtOAc/hexane 7/3). ^**1**^ **H NMR** (400 MHz, CDCl_3_): *δ* 1.00 (3H, t, *J* = 7.4 Hz); 1.05 (3H, t, *J* = 7.4 Hz); 1.43 (3H, d, *J* = 6.6 Hz); 1.50 (3H, d, *J* = 6.5 Hz); 1.90–2.01 (4H, m); 2.09–2.16 (4H, m); 3.64–3.69 (4H, m); 3.76 (6H, s); 3.95 (4H, t, *J* = 5.9 Hz); 4.52–4.57 (2H, m); 4.67–4.76 (4H, m); 5.87–5.92 (2H, m); 6.69 (1H, d x d, *J* = 8.1, 2.6 Hz); 6.75 (1H, d x d, *J* = 8.3, 2.4 Hz); 6.79–6.83 (10H, m); 6.87–6.90 (2H, m); 7.01 (2H, t, *J* = 7.6 Hz); 7.12–7.24 (10H, m); 7.28–7.38 (8H, m). ^**13**^ **C NMR** (100.6 MHz, CDCl_3_): *δ* 9.5; 9.6; 21.8; 22.0; 26.06; 26.08; 27.53; 27.55; 45.1; 45.2; 52.1 (2 x C); 55.7 (2 x C); 65.6 (2 x C); 73.2 (2 x C); 77.5; 77.8; 108.8 (2 x C); 113.8; 114.0; 114.7 (5 x C); 114.9; 115.4 (4 x C); 116.2 (2 x C); 120.4 (2 x C); 120.6; 120.7; 123.9 (2 x C); 126.0 (2 x C); 126.1 (2 x C); 127.9; 128.0; 128.4 (2 x C); 128.5 (2 x C); 129.77; 129.83; 138.6; 138.7; 140.8; 141.0; 143.5 (2 x C); 148.9 (2 x C); 152.9 (2 x C); 153.9 (2 x C); 158.26; 158.32; 162.6 (2 x C); 170.80; 170.83. **IR** (cm^−1^): ν_max_ = 2318, 1748, 1638, 1578, 1508, 1458, 1231. **MS** (70 eV): m/z (%) 595 (M^+^ + 1, 100).

## Supporting Information

Additional supporting information can be found online in the Supporting Information section.

## Funding

This work was supported by Bijzonder Onderzoeksfonds UGent (Grant BOF23/DOC/134).

## Conflicts of Interest

The authors declare no conflicts of interest.

## Supporting information

Supplementary Material

## Data Availability

The data that support the findings of this study are available from the corresponding author upon reasonable request.
