# Supplementary material for: A Convenient Laboratory‐Scale Synthesis Route Toward the PPARα Agonist Pemafibrate
Source: ChemistryOpen. 2026 Mar 25;15(4):e202600009. doi: 10.1002/open.202600009 (PMC13140930; doi:10.1002/open.202600009)

Supporting information

A Convenient Laboratory-scale Synthesis Route toward the PPARα Agonist Pemafibrate

Felien Morlion, Dorien Clarisse, Karolien De Bosscher, Matthias D'hooghe*

Table of contents

NMR spectra of pemafibrate S1

NMR spectra of new compounds S2

NMR spectra of pemafibrate

Pemafibrate **7**

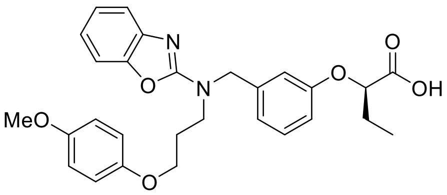

^{1}H NMR (400 MHz, $CDCl_3$)**:**

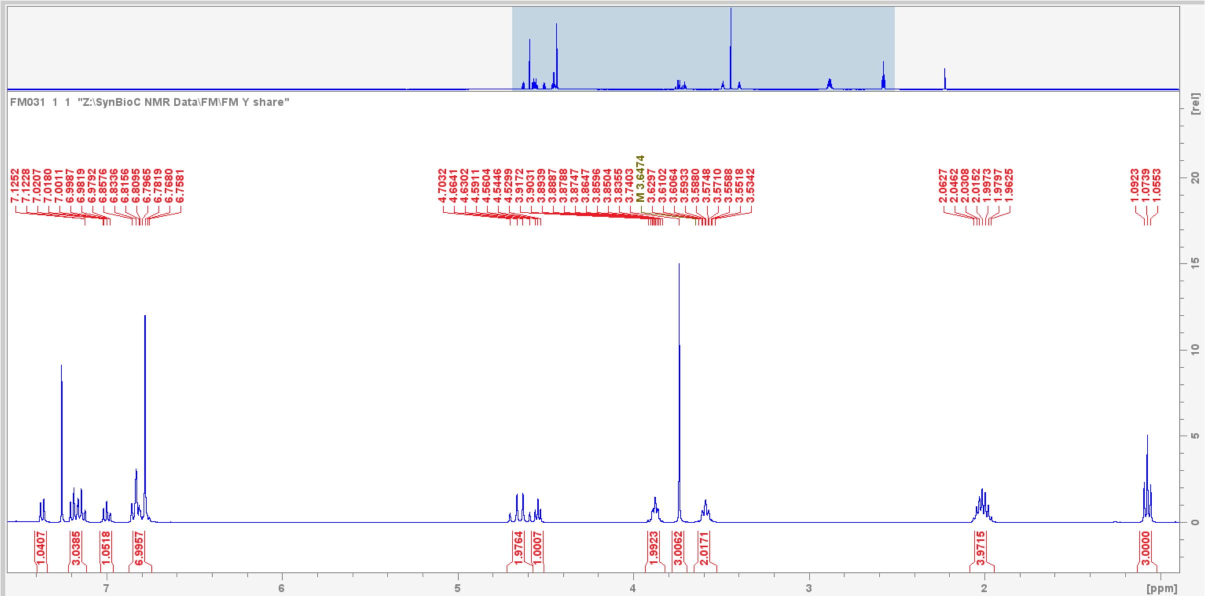

^{13}C NMR (100.6 MHz, $CDCl_3$)**:**

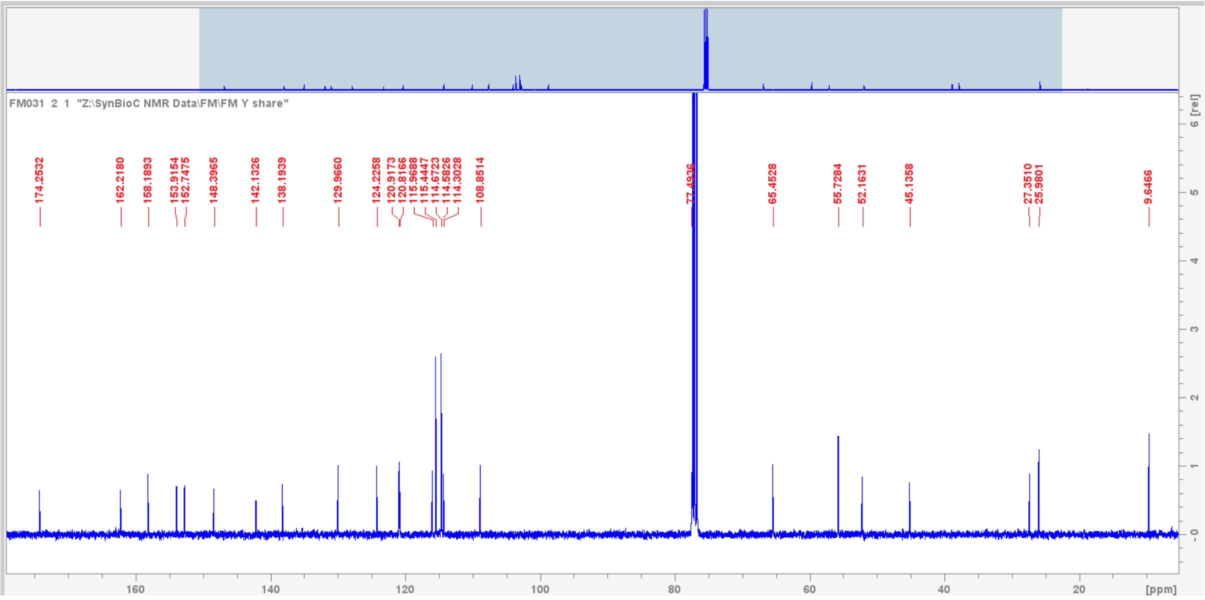

NMR spectra of new compounds

Compound **8**

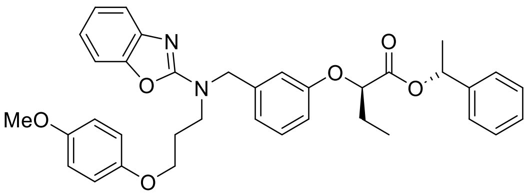

^{1}H NMR (400 MHz, $CDCl_3$)**:**

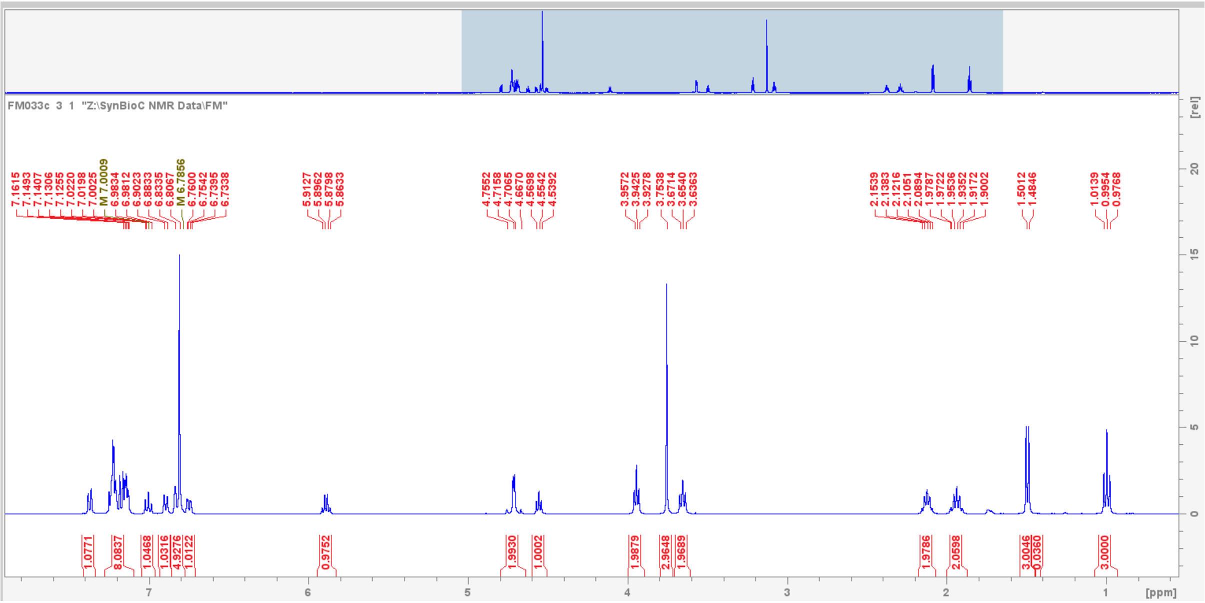

^{13}C NMR (100.6 MHz, $CDCl_3$)**:**

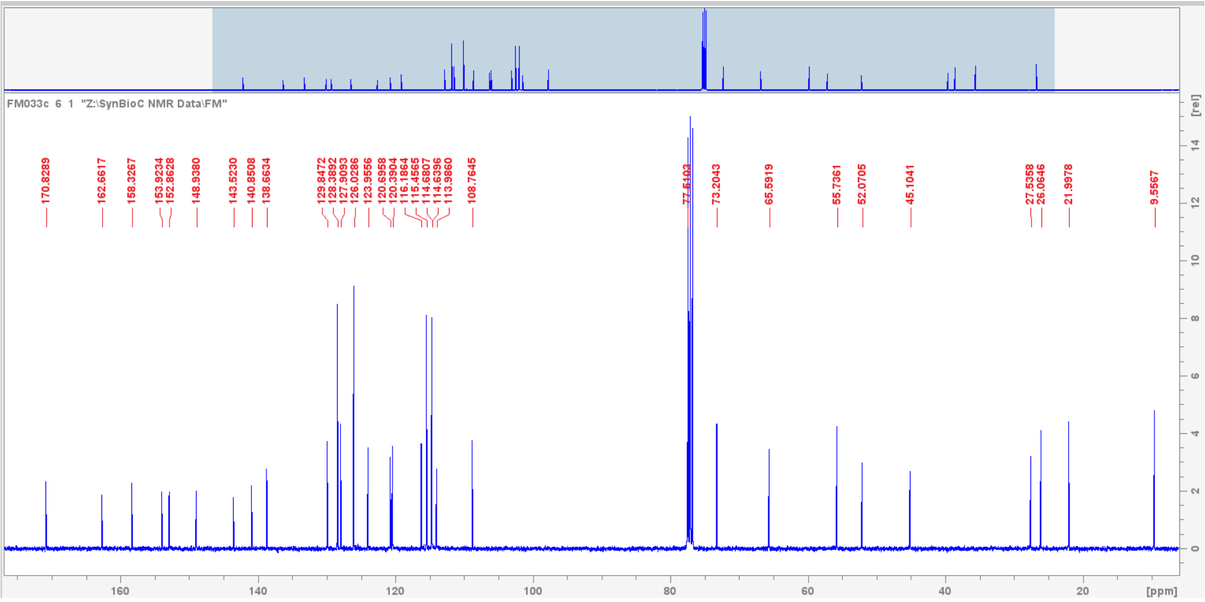

Compound **15**

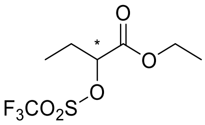

^{1}H NMR (400 MHz, $CDCl_3$)**:**

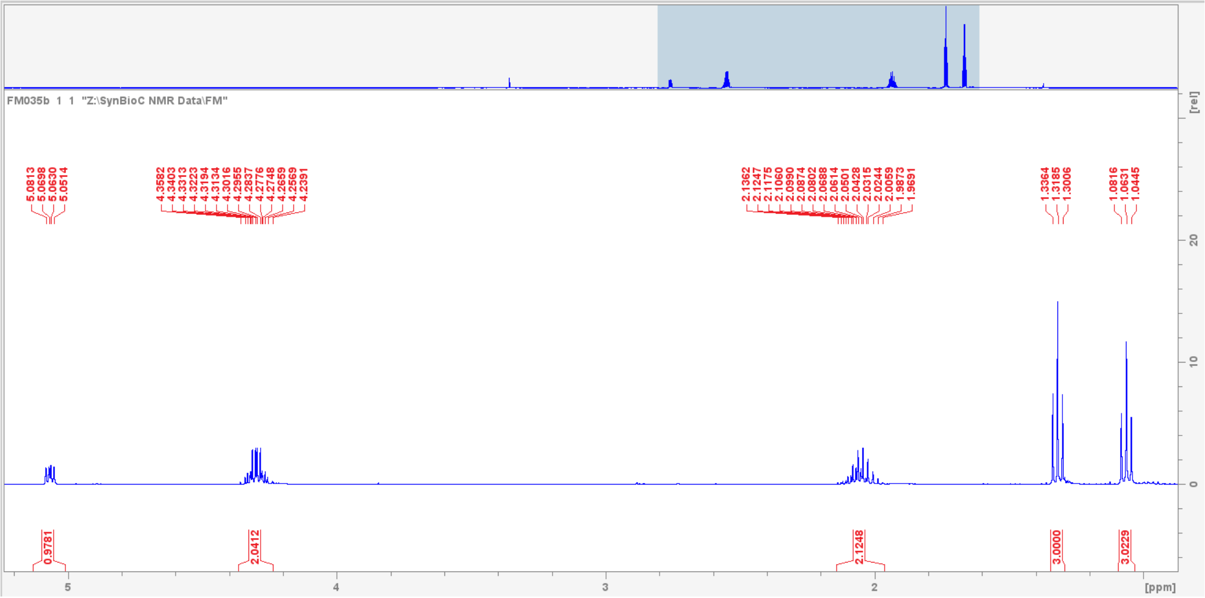

^{13}C NMR (100.6 MHz, $CDCl_3$)**:**

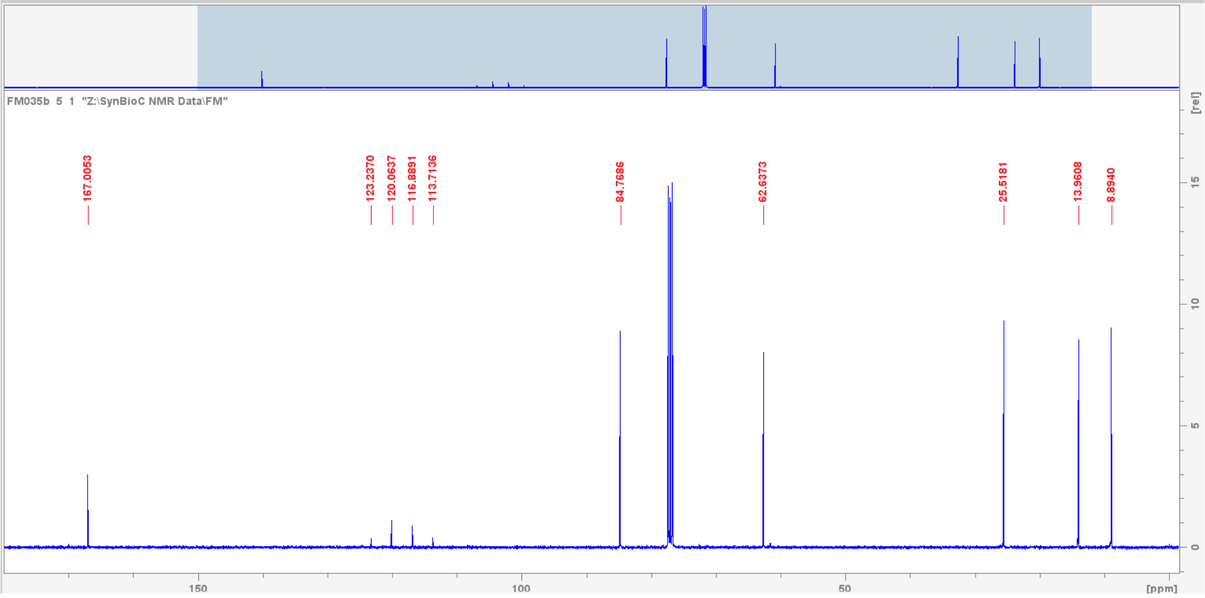

Compound **11**

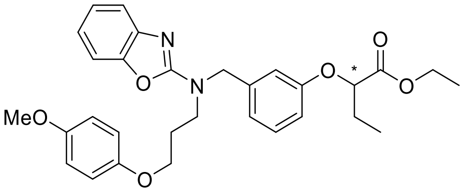

^{1}H NMR (400 MHz, $CDCl_3$)**:**

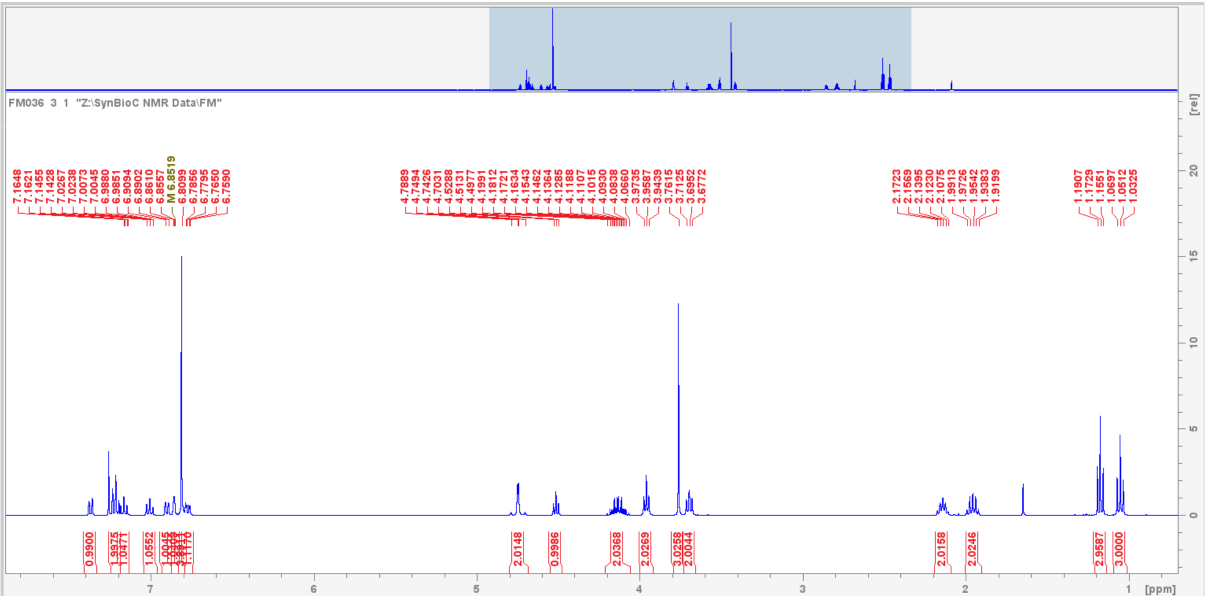

^{13}C NMR (100.6 MHz, $CDCl_3$)**:**

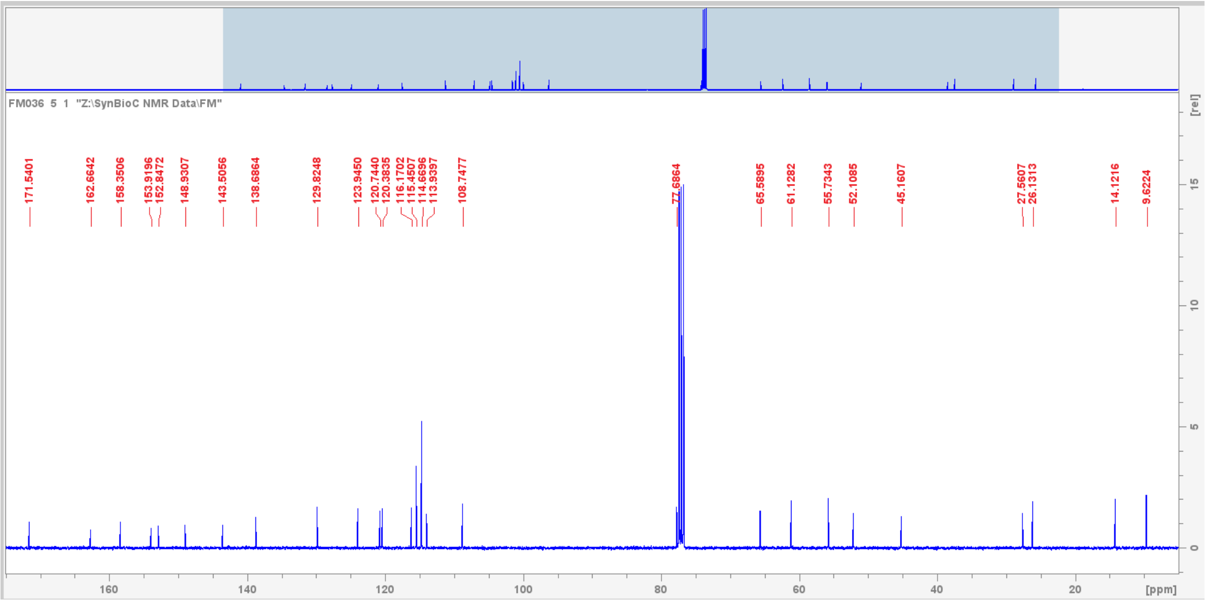

Compound **13**

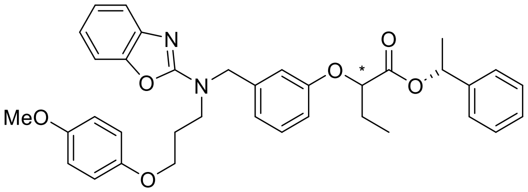

^{1}**H NMR** (400 MHz, $CDCl_3$)**:**

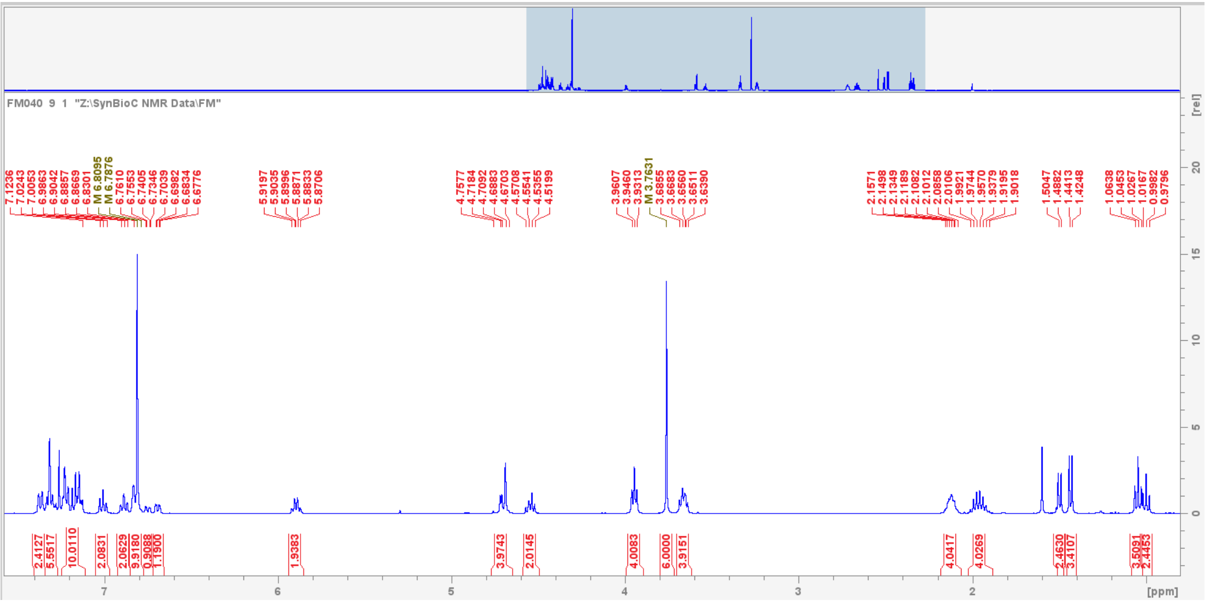

^{13}**C NMR** (100.6 MHz, $CDCl_3$)**:**

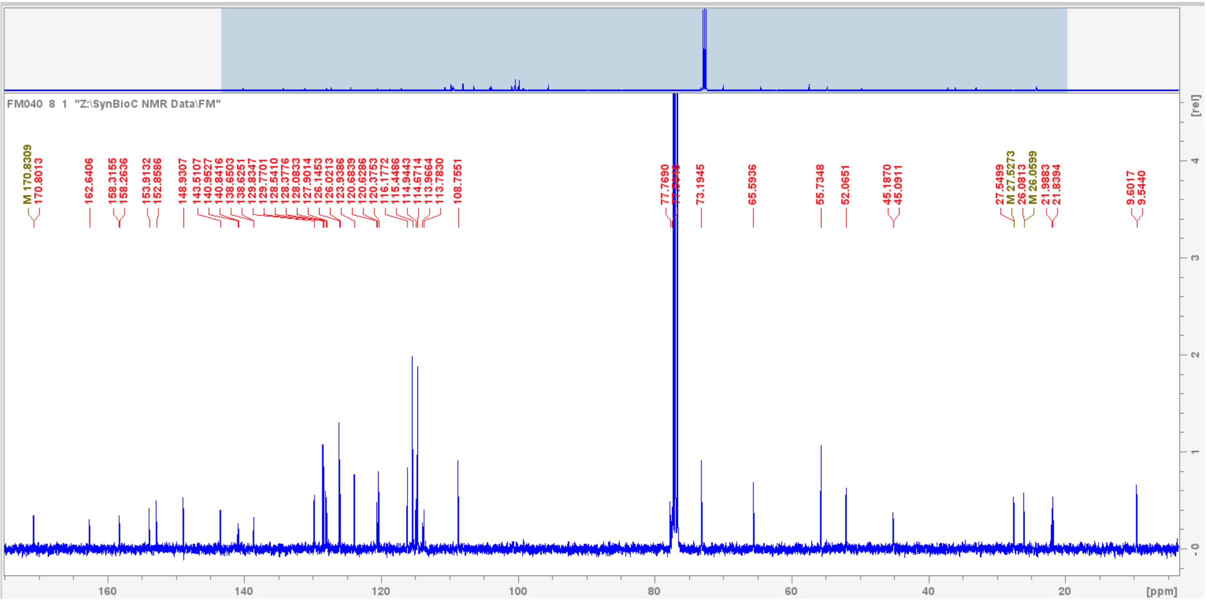

Supplement: Supplementary file 1 — Supplementary Material [file OPEN-15-e202600009-s001.pdf]
